# Photosensitivity responses of *Sagittula stellata* probed by FTIR, fluorescence and Raman microspectroscopy

**DOI:** 10.1039/c9ra03630j

**Published:** 2019-08-30

**Authors:** Marios Papageorgiou, Charalampos Tselios, Constantinos Varotsis

**Affiliations:** Department of Environmental Science and Technology, Cyprus University of Technology Lemesos Cyprus c.varotsis@cut.ac.cy +357 25002802

## Abstract

Raman, fluorescence and FTIR experiments of prestine *Sagittula stellata* and *Sagittula stellata*–metal ion complexes grown in light and in dark were performed to probe the photosensitivity response of the cellular components in the marine bacterium. In the presence of Cu(ii) and Zn(ii) the frequency shifts of PO_2_^−^, C–O–C and C–O–P vibrations indicate metal binding to nucleic acids, carbohydrates and polysaccharides. We assign the observed bands in the 514.1 nm Raman spectra of the prestine *S. Stellata* and of the extracted carotenoids to the C=C and C–C stretching vibrations. The fluorescence excitation–emission matrix (EEM) of *S. stellata* in light, dark and in the presence of metal ions are reported and compared with the Raman and FTIR data. The novel ability of *S. stellata* although heterotrophic, to show light-dependent metal binding ability may be an important feature property that maintains a stable heterotroph–prototroph interaction and a dynamic system.

## Abstract 2

Raman, fluorescence and FTIR experiments of prestine *Sagittula stellata* and *Sagittula stellata*–metal ion complexes grown in light and in dark were performed to probe the photosensitivity response of the cellular components in the marine bacterium.

## Graphical abstract

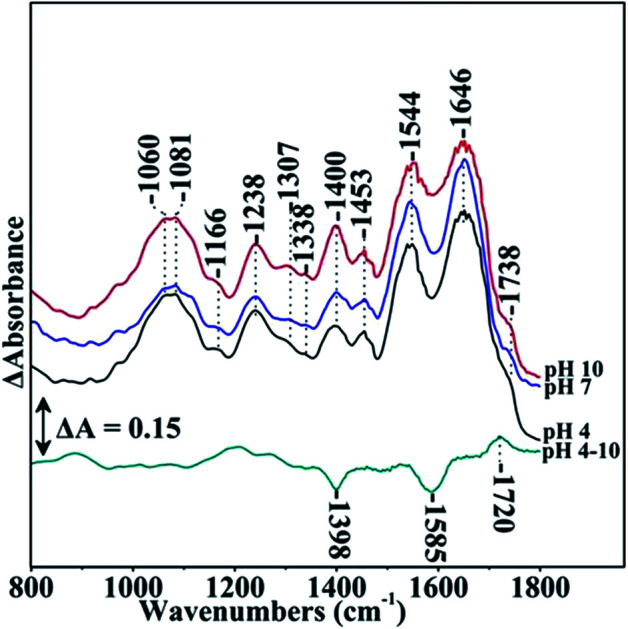

## 1. Introduction

Marine *Roseobacter* clade bacteria (RCB) are one of the most abundant bacterioplanktonic groups in oceans worldwide.^1–3^ RCB are free-living, but they are also often found in epibiotic biofilms on macro-algae and various invertebrates and are key players in the carbon and sulfur cycles and have a variety of mechanisms for obtaining energy. Ecologically, the *Roseobacters* have been proposed to adapt a strategy that takes advantage of the microenvironment with elevated nutrient concentrations from the seawater. Genome analysis has shown that in addition to heterotrophic energy acquisition some members are capable of phototrophy. Light utilization involving bacteriochlorophyll a synthesized by Aerobic Anoxygenic Phototrophic (AAP) *Roseobacters* without producing molecular oxygen is found in fourteen diverse strains.^4^ AAP bacteria may alter current models of the carbon cycle and other biogeochemical processes. The fact that members of the *Roseobacter* lineage can be cultured in the laboratory provides an advantage in efforts to understand their biology and chemistry as well their biogeochemical properties.^5^

*Sagittula stellata* is a heterotrophic member of the alpha proteobacteria *Roseobacter* clade typically found in marine environments responsible for the degradation of cellulose, lignin related compounds and for the oxidation of dimethylsulfide (DMS) to dimethyl sulfoxide (DMSO) that is coupled to ATP synthesis and other organic sulfur compounds typically found in surface.^6,7^ Phototrophic bacteria found in marine environments use DMS which is a volatile organosulfur compound involved in biogeochemical cycling and in climate control, as a carbon or a sulfur source and oxidize it to DMSO. DMS is also oxidized photochemically to DMSO which is present in high concentration in seawater in association with phytoplankton and is degraded in anaerobic environments by methanogens and sulfate reductases.^8^ Light-stimulated DMSO production has led to the hypothesis that phototrophic bacteria may use DMS as an energy source in the environment as observed in pure cultures. There are reports for utilization of DMS as a sulfur source with the aid of light by marine heterotrophic isolates other that *S. stellata* that are able to degrade DMS aerobically. The oxidation of DMS to other compounds in the sea plays an important role in sulfur circulation because the oxidation reduces the release of DMS into the air. There are no data available in the literature regarding the photosensitivity response of *S. stellata* and of the intact and extracted carotenoids.

Trace metals such as iron and copper play important roles in the ocean because they are essential for the growth of marine phytoplankton. These trace metals are also needed for the growth and survival of photosynthetic organisms. Because the speciation of many metals is controlled by pH, a more acidic ocean will alter the bioavailable fractions of these metals. In the surface ocean, the biochemically significant metals for microorganisms are manganese, iron, nickel, cobalt, copper, zinc, and cadmium. On the other hand, metal ions such as Fe(iii), Cu(ii), Mg(ii) and Zn(ii) have been employed in industrial applications and thus are considered as environmental contaminants. There is an increasing concern with respect to their impact and safety in seawaters. Small increases in concentration of normally scarce metals often result in toxic effects to organisms unaccustomed to the higher concentrations.^9,10^ This has been observed with the free form of Cu(ii) which are reported to be toxic to marine phytoplankton. Although iron is known to be used in chlorophyll production and nitrogen fixation, regulating primary productivity and marine biogeochemical cycles, contributes significantly in the degradation of β-carotene, photochemical processes in ocean surface waters produce a number of free radicals that can change the oxidation state of a number of metals. A number of researchers have shown that many biologically significant metals form strong complexes with organic ligands in seawater.^11^ Although a large amount of metal ions products are released in the environment, the mechanism for the interactions between metal ions and surface-seawaters is poorly understood.

Raman spectroscopy is a non-destructive, structure sensitive technique and has been applied in a variety of biological dynamics studies.^12–18^ The FTIR technique offers a unique advantage to probe and monitor at the molecular scale, *in situ*, non-destructively, in real time, and under different hydrated conditions, the biochemical composition of bacteria species attached onto surfaces and the interphase between the surfaces and bacteria and between bacteria.^16,19^ Fluorescence spectroscopy is a non-invasive analytical tool in the study of biomolecules by virtue of its high sensitivity.^20,21^

In this study, we have applied a combination of Raman, fluorescence and FTIR spectroscopies towards establishing a direct method for monitoring the photosensitivity response in the metal binding properties of *S. stellata* and establish the vibrational marker bands for monitoring the functional groups affected by the presence of metal ions at the molecular level. The 514.1 nm excitation Raman data revealed strong scattering from carotenoids and partial and complete photo-degradation in the presence of CuCl_2_ and FeCl_3_, respectively. The dynamics of the fluorescence excitation–emission matrix (EEM) of *S. stellata* in the presence of metal ions are reported, discussed and compared with the FTIR and Raman results. To our best knowledge this is the first application of a comprehensive spectroscopic study involving Raman, FTIR and fluorescence spectroscopies for chemical analysis of the complex bacteria matrix formed by the metal ion binding to *S. stellata* illustrating the potential of the techniques to perform ultrasensitive chemical dynamics analysis of marine bacteria, including the detection of different components and the determination of their relative abundance with the metal ions Cu(ii), Fe(iii), Mg(ii), and Zn(ii).

## 2. Experimental

### 2.1. Sample preparation

*Sagitula stellata* bacteria were purchased from the Deutsche Sammlung von Mikroorganismen und Zellkulturen GmbH (DSMZ) Company and maintained and pre-cultured aerobically at 25 and 28 °C in Difco Marine Broth 2216 medium in the dark and in light (70 W m^−2^). The highest concentration of bacteria cells was observed in the third day of cultivation. The concentration of the cells was measured every day at 600 nm by an Agilent Technologies Cary 60 UV-Vis. The bacteria cells in the third day of cultivation were centrifuged for 7 min at 13 200 rpm. The supernatant was removed and sterilized and the procedure was repeated twice. All samples were prepared in phosphate buffer (10 mM) and the pH was adjusted to 4.0, 7.0 and 10.0 for FTIR analysis.

Two bacteria cultures of *S. stellata* were grown at 28 °C in dark and light conditions respectively. The optical density (OD at 600 nm) was between 0.8 and 1.0 for each sample when the cells were harvest for carotenoids extraction according to published procedures.^22,23^ 10 mL of each culture were transferred in 15 mL tube and centrifuged at 4000 × *g* rpm (4 °C) for 4 min. The supernatant was discarded and the remaining bacteria were centrifuged again at 11 000 × *g* rpm (4 °C) for 1 min. 200 μl of pre-chilled methanol was added on the pellet and the sample was vortexed and sonicated (10 s each). The procedure repeated twice with the addition of 200 μL of acetone and dichloromethane. The samples were incubated on ice for 2 min and then centrifuged at 11 000 × *g* rpm (4 °C) for 4 min. The procedure was performed under dim light. The supernatant was collected and used for the UV-Vis and Raman experiments.

### 2.2. FTIR spectroscopy

The *S. stellata*–CuCl_2_, –FeCl_3_, –MgCl_2_ and –ZnCl_2_ metal complexes (30 mM) were incubated for 5 days with gentle stirring. The FTIR spectra were recorded using a Vertex 70v FTIR spectrometer (BRUKER) equipped with a photovoltaic MCT detector. The sample compartment was purged with nitrogen gas. The bacterial solutions were centrifuged and the supernatant was carefully removed. The precipitated was placed on silver fluoride window and let to dry out and form a film. The spectral signal was averaged from 100 scans at 4 cm^−1^ resolution, and collected from 800–4000 cm^−1^.

### 2.3. Raman spectroscopy

The 514.1 nm excitation Raman spectra of *S. stellata* and of the CuCl_2_, FeCl_3_, MgCl_2_ and ZnCl_2_ complexes (30 mM) were performed with a iHR550 Raman spectrometer equipped with Olympus BX41 microscope and a Synapse CCD Detector (Horiba), controlled by Syner JY software (Horiba). The 514.1 nm excitation laser beam was provided by a Coherent Sapphire laser. The laser power incident on the sample was 20 mW. The total accumulation time for each measurement was 5 minutes.

### 2.4. Fluorescence spectroscopy

The Cary Eclipse Fluorescence Spectrophotometer (Agilent Technologies, USA) controlled from a PC using the Agilent Technologies WinFLR software (version 1.2) was used for obtaining the fluorescent signals. Fluorescence spectra of prestine and in the presence of CuCl_2_ and FeCl_3_, (3.5 mM, pH 7) *S. stellata* samples after one and five-days incubation under gentle stirring were measured using a clear four-sided quartz cuvette in the spectrophotometer sample compartment. The excitation and emission slit widths for both the excitation and emission monochromators were set with the value of 5.0 nm and the scan speed was 120 nm min^−1^. The Excitation Emission Matrix (EEM) measurement and processing were generated by scanning emission spectra from 230 nm to 550 nm at 1 nm intervals, with 5 nm increments of the excitation wavelengths from 220 nm to 400 nm.

## 3. Results and discussion

### 3.1. Fourier transform infrared of *S. stellata*

The chemical composition of marine microorganisms have been monitored by FTIR spectroscopy providing unique insight for studying the bacteria dynamics. The typical FTIR spectrum consists of the vibrations arising from amide I and II, the lipids and phospholipids, the polysaccharides, the water, and finally those from nucleic acids. Proteins mainly are identified by the N–H stretching and the vibrations of the peptide linkage. Peaks at 1646 and 1545 cm^−1^ are attributed to amide I (backbone amide C=O stretching vibration) and amide II bands (out-of-phase combination of the NH in plane band and the CN stretching vibrations) whereas the peaks ranging from 900 to 1200 cm^−1^ have been attributed to the C–O stretch of bacterial polysaccharides and phosphate (symmetric <svg xmlns="http://www.w3.org/2000/svg" version="1.0" width="10.400000pt" height="16.000000pt" viewBox="0 0 10.400000 16.000000" preserveAspectRatio="xMidYMid meet"><metadata> Created by potrace 1.16, written by Peter Selinger 2001-2019 </metadata><g transform="translate(1.000000,15.000000) scale(0.011667,-0.011667)" fill="currentColor" stroke="none"><path d="M80 1160 l0 -40 40 0 40 0 0 -40 0 -40 40 0 40 0 0 -40 0 -40 40 0 40 0 0 -40 0 -40 40 0 40 0 0 -40 0 -40 40 0 40 0 0 -40 0 -40 40 0 40 0 0 -40 0 -40 40 0 40 0 0 80 0 80 -40 0 -40 0 0 40 0 40 -40 0 -40 0 0 40 0 40 -40 0 -40 0 0 40 0 40 -40 0 -40 0 0 40 0 40 -40 0 -40 0 0 40 0 40 -80 0 -80 0 0 -40z M560 520 l0 -40 -40 0 -40 0 0 -40 0 -40 -40 0 -40 0 0 -40 0 -40 -40 0 -40 0 0 -40 0 -40 -40 0 -40 0 0 -40 0 -40 -40 0 -40 0 0 -40 0 -40 -40 0 -40 0 0 -40 0 -40 80 0 80 0 0 40 0 40 40 0 40 0 0 40 0 40 40 0 40 0 0 40 0 40 40 0 40 0 0 40 0 40 40 0 40 0 0 40 0 40 40 0 40 0 0 80 0 80 -40 0 -40 0 0 -40z"/></g></svg> PO_2_ stretching).

**Fig. 1:**
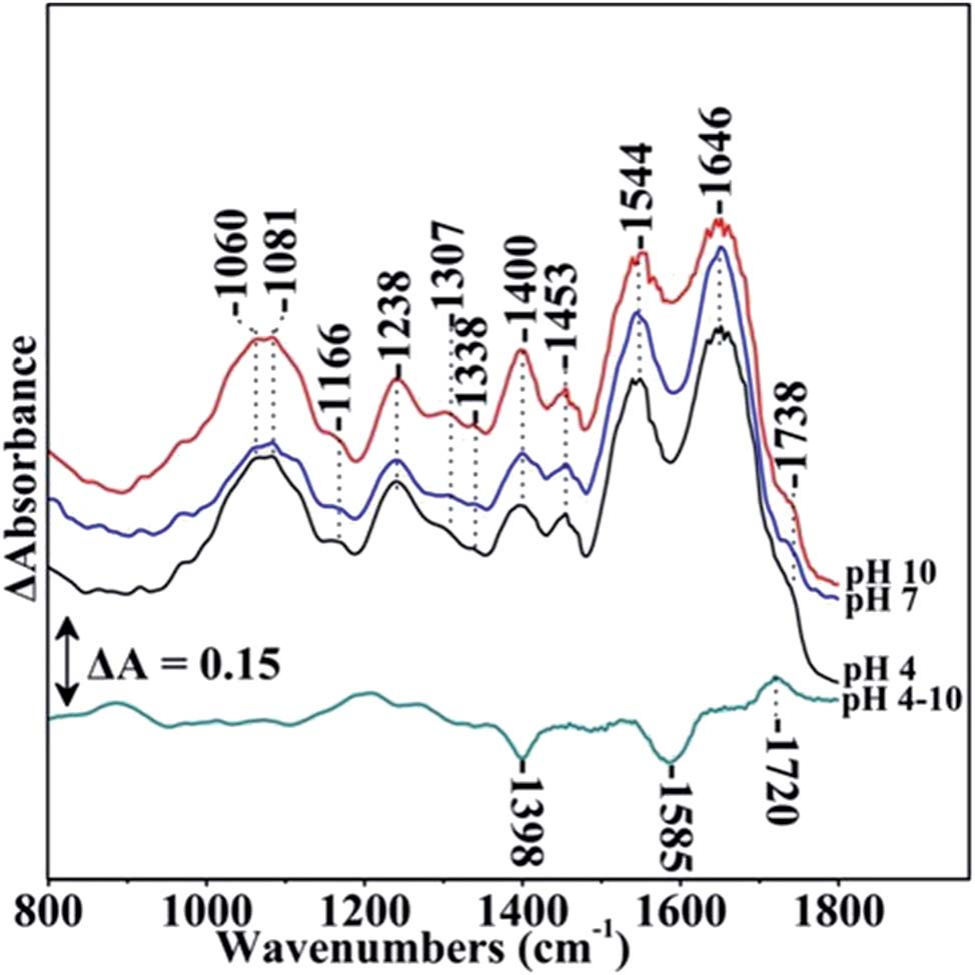
Fourier transform infrared spectroscopic spectra of *S. stellata* in the pH 4–10 range. The spectral range was 800–1800 cm^−1^ and the spectral analysis was 4 cm^−1^.

Fig. 1 shows the FTIR spectra of *S. stellata* in the pH 4–10 range. The major bands are at 1645 and 1545 cm^−1^ which are attributed to the amide I and amide II bands arising from the polypeptide backbone of proteins. In all pH examined a small peak is observed at 1738 cm^−1^ which is assigned to the ester C=O bonds of lipids. Ester moieties (C–OR vibration) contributes also to the broad peak at 1243 cm^−1^ together with phosphate groups (asymmetric PO_2_^−^ stretching) and protein backbone (amide III band). The polysaccharide fraction (alcoholic C–OH vibration) and phosphate (symmetric PO_2_ stretching) account for the signals at 1067 and 1087 cm^−1^. The bands at 1400 and 1454 cm^−1^ are due to methylene and methyl bending modes and/or to deprotonated carboxylates (symmetric stretching vibration). The signal at 1305 cm^−1^ is not assigned to a specific group but to generic functional groups in agreement with that observed in the FTIR spectra of EPS from bacterial layers.^23–25^ The difference spectrum pH 4 minus pH 10 shows negative peaks at 1398 and 1585 cm^−1^ and a positive peak at 1720 cm^−1^ indicating the presence of antisymmetric and symmetric COO moieties at pH 10 and also protonated COOH groups at pH 4.

**Fig. 2:**
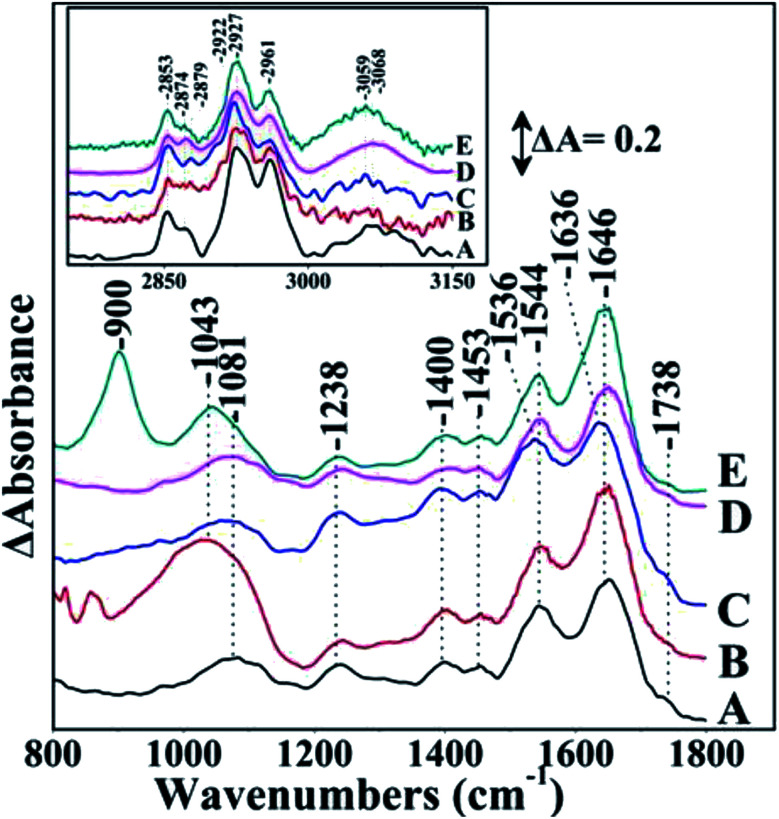
Fourier transform infrared spectrum A of *S. stellata*, spectra B, C, D, and E are those of *S. stellata* after 5 days incubation with 30 mM CuCl_2_, FeCl_3_, MgCl_2_ and ZnCl_2_, respectively. The spectral range was 800–1800 and 2000–3150 cm^−1^ (inset) and the spectral resolution was 4 cm^−1^.

Fig. 2, shows the FTIR spectra of *S. stellata* in the presence of 30 mM CuCl_2_, FeCl_3_, MgCl_2_ and ZnCl_2_. Small changes are observed in the FTIR spectra by the presence of metal ions. The ratio of amide I (1646 cm^−1^)/amide II (1545 cm^−1^) is 1.77 in the prestine *S. stellata* and 1.9, 1.6, 1.7 and 2.5 in the CuCl_2_, FeCl_3_, MgCl_2_ and ZnCl_2_ complexes. On the same line, the ratio of the characteristic frequencies of lipids at 2927/2961 cm^−1^ is 1.47 in the prestine bacterium and 2.15, 2.3, 3.65 and 2.55 in the CuCl_2_, FeCl_3_, MgCl_2_ and ZnCl_2_ complexes with the bacterium. It should be noted that in the case of FeCl_3_ the amide I and amide II were observed at 1636 and 1536 cm^−1^, respectively. These results strongly indicate that there is preferential adsorption of *S. stellata* on metal ions. Furthermore, the stretching vibrations of PO_2_ exhibit significant changes in frequency/intensity after complexation with the metal ions. This result indicates that the electronic density of the phosphorous atom is weakened upon complexation. Of note is the emergence of a new band located at 1043 cm^−1^ that is consistent with P–O–M^+^ bonds. Emergence of the new band is consistent with inner-sphere complexation of bacteria phosphate groups which mostly derive from phosphodiesters of proteins and nucleic acids with the metal ions. In the case of Zn and Cu the free P=O is absent and only the new band is observed at 1043 cm^−1^. Therefore, both the amide groups in proteins and phosphate groups in phosphodiester bridges of nucleic acids contribute to the metal ion complexation from *S. stellata*. Therefore, the FTIR results suggest that electrostatic interaction play an important role in the formation of inner-sphere complexes between bacteria and M^+^.^26,27^

**Fig. 3:**
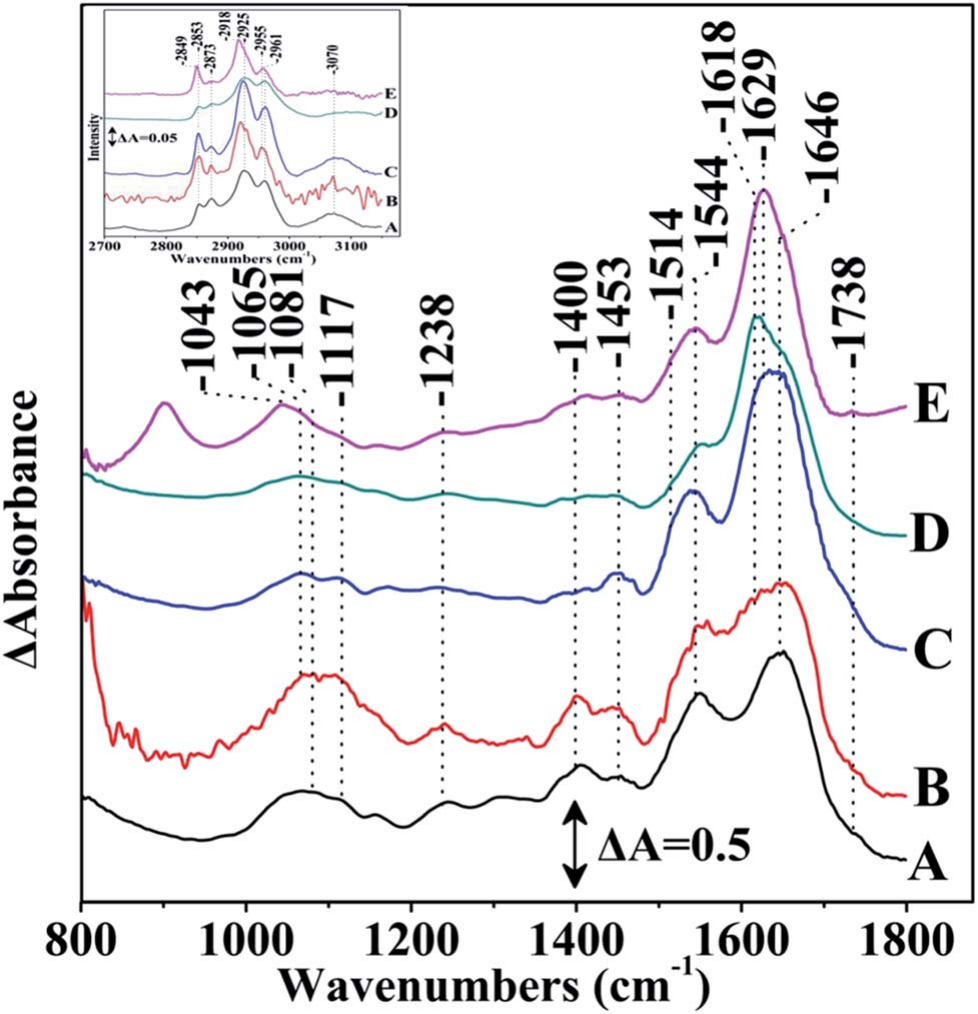
Fourier transform infrared spectra of *S. stellata* samples exposed to 70 W m^−2^ of light for 5 days. Spectrum A is of *S. stellata*, spectra B, C, D, and E are taken after 5 days of incubation of the bacterium with 30 mM CuCl_2_, FeCl_3_, MgCl_2_ and ZnCl_2_, respectively. The inset shows the corresponding data in the 2700–3100 cm^−1^ region. The spectral range was 800–1800 cm^−1^.

In Fig. 3 we present the FTIR spectra under the same experimental conditions as those presented in Fig. 3 with the exception that the samples were grown in the presence of 70 W m^−2^ of light and left in light in the presence of metal ions for five days. A close inspection of the data reveals no significant differences of the prestine *S. stellata* in light and in dark. However, in the presence of metal ions there are significant frequency shifts and intensity differences in the region of the amide I and amide II as well in the region of the lipids demonstrating the photo-induced conformational changes observed in the FTIR spectra that affect the dynamics of the metal ions in their reactions with *S. stellata*. A close inspection and comparison of the data reveals that in the presence of light the ratio of amide I (1646 cm^−1^)/amide II (1545 cm^−1^) is 1.43 in the prestine bacterium is decreased from that observed in the dark and is 0.62 (decreased), 2.18 (increased), 3.03 (increased) and 2.78 (decreased) in the CuCl_2_, FeCl_3_, MgCl_2_ and ZnCl_2_ bacterium complexes, respectively. On the same line, the ratio of the characteristic frequencies of lipids at 2925/2961 cm^−1^ is 2.77 (increased) in the prestine bacterium and 2.97 (slightly increased), 2.97 (decreased), 1.89 (decreased) and 4.95 (increased) in the CuCl_2_, FeCl_3_, MgCl_2_ and ZnCl_2_ bacterium complexes, respectively. The FTIR spectra of the prestine *S. stellata* grown in light and in dark, show no difference between the major protein and lipid bands. However, in the presence of metal ions the FTIR spectra are seriously altered and are metal-dependent.

### 3.2. Raman investigation of *Sagittula stellata*

**Fig. 4:**
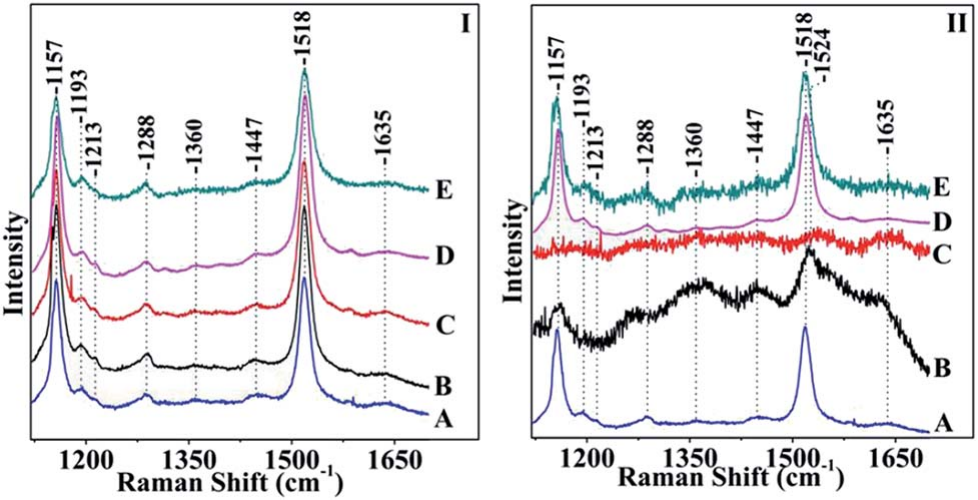
Raman spectra of *S. stellata* exposed to room-light (panel I) and to 70 W m^−2^ white light (panel II) in the presence of metal ions. Spectra A are in the absence of metal ions, spectra B, C, D, and E are those taken after five days of incubation with 30 mM CuCl_2_, FeCl_3_, MgCl_2_ and ZnCl_2_, respectively. The excitation wavelength was *λ*_exc_ = 514.1 nm and the laser power incident on the sample was 20 mW. The total accumulation time for each spectrum was 5 min.

Fig. 4 shows the Raman spectra of *S. stellata* grown under normal room-light (panel I) and under of 70 W m^−2^ white light for five days (panel II). Spectra A are in the absence of metal ions. Spectra B, C, D, and E are those taken after five days incubation with 30 mM CuCl_2_, FeCl_3_, MgCl_2_ and ZnCl_2_, respectively. Carotenoids which are widespread in microbial communities and are present in all phototrophic microorganisms and in certain heterotrophic have strong Raman signals in a non-resonant mode.^28^ With 514.1 nm excitation which is in resonance with the lowest allowed optical transition S_0_–S_2_ transition information regarding the nature of the C–C and C=C bonds is possible. The Raman spectra shown in panel I are characterized by peaks at 1157 cm^−1^ due to C–C stretching of carotenoids, the 1193 cm^−1^ to C–C stretching of carotenoids, the peak at 1447 cm^−1^ due to asymmetric methyl deformation and the peak at 1518 cm^−1^ due to C=C stretching. The C–H rocking is the internal coordinate that dominates the modes between 1250 and 1400 cm^−1^ leading to an asymmetric peak at 1288 cm^−1^. The position of the Raman signal at 1518 cm^−1^ suggests that the major carotenoid present may have 10 conjugated double bonds in its structure as deduced by comparing it with the Raman spectra of other carotenoids like spheroidene (*N* = 10) which has 10 conjugated double bonds and has a Raman signal at 1520 cm^−1^.^29^

Comparison of the Raman spectra in panel I demonstrates that the presence of metal ions does not affect the properties of the carotenoids in *S. stellata* when the microorganism is grown under normal conditions. In panel II, spectrum B shows that the major C–C at 1157 cm^−1^ has lost intensity whereas the C=C stretching frequency shows a significant 6 cm^−1^ upshift and appears as a broad peak at 1524 cm^−1^. Spectrum C shows that all characteristic features, in contrast to those observed in panel I have disappeared illustrating the effect of FeCl_3_ to the carotenoids. Spectra D and E are very similar with spectrum A indicating that MgCl_2_ and ZnCl_2_ have minimum interaction with the carotenoids in *S. stellata*. A relatively weak C=C π-coordination bond may cause conformational changes and/or metal binding at each C=C site, which would be important for assembling metal atoms in a convergent manner. A wavelength excitation frequency shift of the *ν*(C=C) has been reported demonstrating the presence of two modes which exhibit different excitation profiles in the region of the allowed electronic S_0_–S_2_ transition with maxima that are separated by *ca.* 1300 cm^−1^.^28^ The frequency shift we have observed is attributed to conformational heterogeneity induced by the presence of the metal ion resulting to a slight elongation of the C=C bonds without affecting the C–C bonds as a possible explanation for this shift. Carotenoids (Car) are UV radiation protectants due to their antioxidant and light harvesting properties. Most of the bacteria that grow in regions of extreme sunlight exposure are equipped with carotenoids to survive against large doses of UVA radiation exposure. Oxidation of β-carotene generates Car radical cations (Car˙^+^) which reacts with O_2_ to produce the epoxide form of Car which is subsequently undergoes cleavage to form apo-carotenals and carotenones.^30^ The radical species generated *via* FeCl_3_ and the electrochemical oxidation have been reported. The Raman data indicate that only in the presence of light there is strong evidence for oxidative degradation in the presence of Cu and Fe. It is expected that the light induced oxidative degradation exhibit different kinetics of photodegradation by light irrespective of its interaction with other cellular components.

**Fig. 5:**
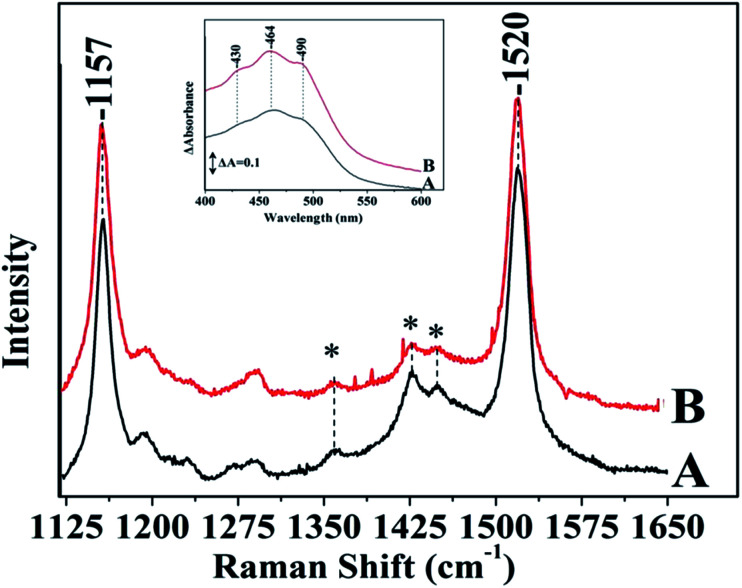
Raman 514.1 nm excitation spectra of carotenoid extracted from *S. stellata* cultures grown in dark (spectrum A) and in light (spectrum B). The inset shows the corresponding UV-Vis spectra. The excitation wavelength was *λ*_exc_ = 514.1 nm and the laser power incident on the sample was 20 mW. The total accumulation time for each spectrum was 20 min. The solvent peaks are denoted by asterisk.

Fig. 5 shows the 514.1 nm excitation Raman spectra of the isolated carotenoids from *S. stellata* grown in dark (spectrum A) and in light (spectrum B). The most intense *ν*_1_ band appears at 1520 cm^−1^ arises from the stretching vibrations of C=C double bonds. The positions of *ν*_1_ depends on the length of the π-electron conjugated chain and the *cis*–*trans* isomerization.^28^ There is also a correlation between the position of the *ν*_1_ Raman band and the polarizability of the solvent. The *ν*_2_ band is constituted by a cluster of contributions around 1157 cm^−1^ that arises from the stretching vibrations of C–C single bonds coupled with C–H in-plane bending modes. The UV-Vis spectra of the carotenoid extracted from *S. stellata* cultures grown in dark (spectrum A) and in light (spectrum B) are shown in the inset. The S_0_–S_2_ transition of carotenoids exhibit a characteristic three-peak structure in the 400–450 nm region corresponding to the lowest three vibronic bands of the electronic transition S_0_–S_2_, termed 0–0, 0–1 and 0–2.^30^ The position of the absorption transition of carotenoid molecules depends on the solvent polarizability and has been studied extensively.^30^ There is a consensus that as the number of double bands of the polyene backbone increases, the wavelength maximum of the S_0_–S_2_ optical transition increases, and the molecule is easier to oxidize. Therefore, by decreasing the number of conjugated double bonds the carotenoid will be more difficult to oxidize. The energy of the S_0_–S_2_ transition and the measurement of the *ν*_1_ Raman band can give accurate values for the conjugation chain length *N* of the carotenoid. Based on the available energies of the S_0_–S_2_ and the *ν*_1_ at 1520 cm^−1^ reported in the literature and ours (data not shown) of commercially available carotenoids we conclude that the isolated species is a carotenoid having 10 conjugated double bonds like spheroidene (*N* = 10) in its structure and its properties are independent of the whether *S. stellata* was grown in the dark or light. Comparison of the data of the isolated carotenoid shown in Fig. 5 with those of the intact carotenoids we conclude that there is a great similarity in the entire conjugated π-systems and the protein environment has little effect on the conformation of carotenoids in *S. stellata.*

### 3.3. Fluorescence of *S. stellata*

**Fig. 6:**
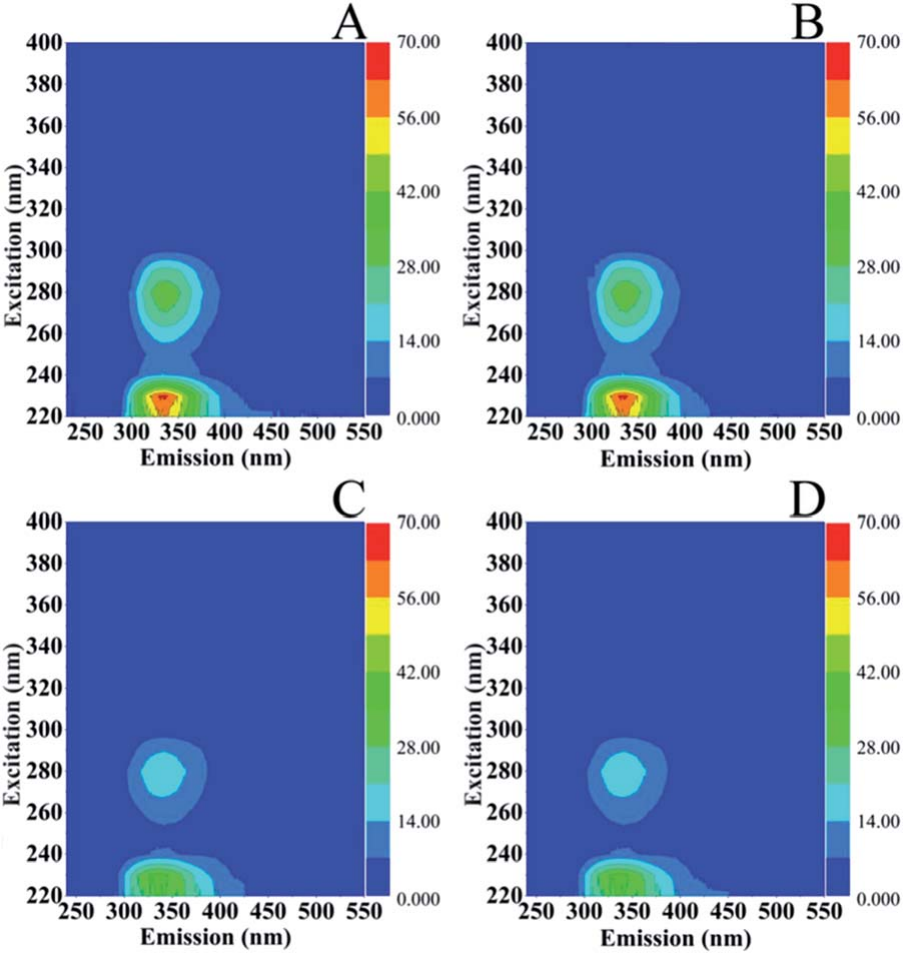
Excitation (*E*_x_)–emission (*E*_m_) matrices (EEMs) of *S. stellata* incubated in the dark (spectra A and B) and in light (spectra C and D). Incubation time for *S. stellata* in spectra A and C was one day and for spectra B and D five days.

There is consensus on the characteristic excitation and emission (EEM) fluorescence properties of marine microorganisms.^31^ The region around aromatic protein (AP) substances is observed at *E*_x_/*E*_m_ = 220–230/340–350 nm with characteristic *E*_x_/*E*_m_ = 220–230/310–360 nm that resemble tyrosines (Y)-, tryptophans (W)- and phenylalanine (Phe)-like fluorescence, protein like fluorescence is observed at *E*_x_/*E*_m_ = 275/305–340 nm, soluble microbial products (SMP) at *E*_x_/*E*_m_ = 280–285/340–350 nm and of the corresponding humic-like substances fluorescence at *E*_x_/*E*_m_ = 290–360/370–460 nm and *E*_x_/*E*_m_ = 440/500 nm. In Fig. 6, the EEM of *S. stellata* grown in the dark (spectra A and B) have *E*_x_/*E*_m_ = 220–245/330–440 nm characteristic of AP and 260–280/300–360 nm characteristic of SMP and humic-like substances. *S. stellata* samples exposed to 70 W m^−2^ of light show characteristic *E*_x_/*E*_m_ = 220–235/300–430 nm which are affected when compared to the corresponding *E*_x_/*E*_m_ of the samples grown in the dark. In addition the intensities at *E*_x_/*E*_m_ = 230/330 nm are reduced and the *E*_x_/*E*_m_ = 235–250/320–350 nm show, in contrast to the samples grown in the dark (spectra A and B) no fluorescence features. On the other hand, the *E*_x_/*E*_m_ = 260–290/300–350 nm are similar to those observed in the samples grown in the dark. Therefore, the regions around AP substances and SMP are affected by light.

**Fig. 7:**
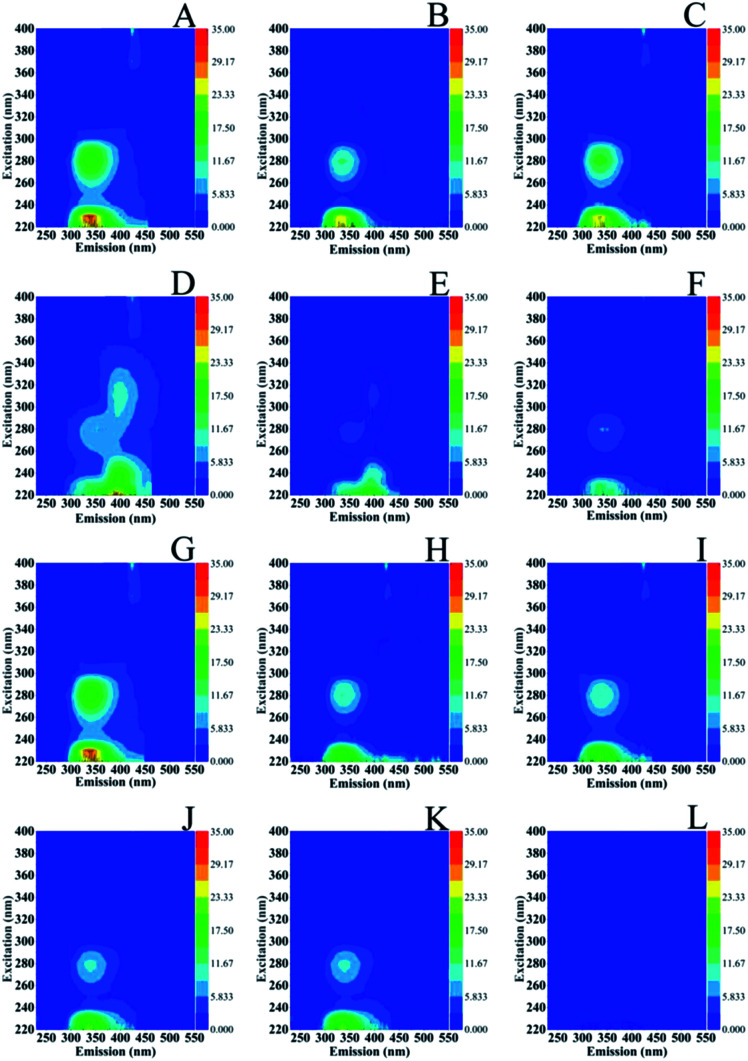
Excitation (*E*_x_)–emission (*E*_m_) matrices (EEMs) of *S. stellata* incubated with 3.5 mM Cu(ii) (spectra of A–C) and 3.5 mM Fe(iii) (spectra D–F) in the dark for one day (spectra A and D), three days (spectra B and E) five days (spectra C and F). Spectra G–L are of *S. Stellata* incubated in light with 3.5 mM Cu(ii) (spectra G–I) and 3.5 Fe(iii) for one day (spectra G and J), three days (spectra H and K) and five days (spectra I and L).

Fig. 7 shows the excitation (*E*_x_)–emission (*E*_m_) matrices (EEMs) of *S. stellata* in the dark (spectra A–F) and in light (spectra G–L) incubated with 3.5 mM Cu(ii) (spectra of A–C) and 3.5 mM Fe(iii) (spectra D–F) for one day (spectra A and D), three days (spectra B and E) five days (spectra C and F). Spectra G–L are of *S. stellata* incubated in light with 3.5 mM Cu(ii) (spectra G–I) and 3.5 Fe(iii) (spectra J–L) for one day (spectra G and J), three days (spectra H and K) and five days (spectra I and L). All the EEM spectra of *S. stellata* (spectra A–C) in the presence of Cu(ii) in the time period of one to three days show small intensity variations when compared with those of prestine *S. stellata* shown in Fig. 6 (spectra A and B). The analogous EEM spectra in the presence of Fe(iii) (spectra D and E) show distinct differences when compared with those of Fig. 6 (spectra A and B). In the spectra of the *S. stellata*–Fe(iii) complexes taken in the first day of incubation changes in the regions around AP substances at *E*_x_/*E*_m_ = 220–230/340–350 nm and SMP at *E*_x_/*E*_m_ = 280–285/340–350 are evident and are retained in the spectra of the complexes taken in the third and fifth day of incubation with Fe(iii). In the presence of light, spectra (G–I) resemble those observed in the dark (spectra A–C) for the whole time of incubation with Cu(ii). The *E*_x_/*E*_m_ in spectra J–L are similar to those observed in the absence of light, however in the fifth day of incubation with Fe(iii) in light both the *E*_x_/*E*_m_ of AP and SMP are affected by shown frequency shifts and intensity changes. It should be noted that with concentrations of CuCl_2_ and FeCl_3_ less than 3.5 mM there is no evidence for any changes in the EEMs.

## 4. Conclusions

Certain biomolecules, such as proteins excreted from bacteria can form aggregates *via* cross-linking due to photo-oxidation.^32,33^ On the other hand, sunlight can inhibit or disrupt the processes of marine colloids *via* cleavage of high molecular weight compounds into smaller, less stable fragments.^34^ Raman, fluorescence and FTIR spectroscopy have been shown to be critical techniques for obtaining information about the structural changes of bacteria components following proton and metal cation (CuCl_2_, FeCl_3_, MgCl_2_ and ZnCl_2_) uptake. The great stability of the *S. stellata* allowed successfully running FTIR, fluorescence and Raman experiments in the presence of metal cations, with the exception of the Fe and Cu–bacteria complexes under 514.1 nm photo-stationary conditions. With this approaches a comprehensive spectroscopic investigation for the metal binding properties described in this paper is of profound importance because it allows evaluating the strength of the bacteria in bioremediation of heavy metal contamination. Photodegradation of carotenoids has been observed even in the absence of FeCl_3_.^30^ In the presence of FeCl_3_ and CuCl_2_, the ferric and cubric photocatalytic degradation provides new insights in the oxidation of carotenoids. Whether the formation of humic-like substances by the cubric photocatalytic degradation and the photodegradation of carotenoids are related to each other and the dynamics of the light responses of heterotrophic and phototrophic *Roseobacter* clade bacteria are under investigation in our laboratory.^3,35^

## Conflicts of interest

There are no conflicts to declare.

## Supplementary Material

## References

[cit1] Gonzalez J., Moran M. (1997). Appl. Environ. Microbiol..

[cit2] Buchan A., Gonzalez J. M., Moran M. A. (2005). Appl. Environ. Microbiol..

[cit3] Wagner-Döbler I., Biebl H. (2006). Annu Rev Microbiol.

[cit4] Allgaier M., Felske A., Wagner-do I. (2003). Appl. Environ. Microbiol..

[cit5] Luo H., Moran M. A. (2014). Microbiol. Mol. Biol. Rev..

[cit6] Boden R., Murrell J. C., Schafer H. (2011). FEMS Microbiol. Lett..

[cit7] Ding Y. X., Chin W. C., Rodriguez A., Hung C. C., Santschi P. H., Verdugo P. (2008). Mar. Chem..

[cit8] Fuse H., Takimura O., Murakami K., Yamaoka Y. (2010). Appl. Environ. Microbiol..

[cit9] Sunda W. G., Huntsman S. A. (1995). Limnol. Oceanogr..

[cit10] Das S., Mangwani N. (2015). Oceanologia.

[cit11] Decho A. W., Gutierrez T. (2017). Front. Microbiol..

[cit12] Pinakoulaki E., Varotsis C. (2003). Biochemistry.

[cit13] Pinakoulaki E., Varotsis C. (2008). J. Inorg. Biochem..

[cit14] Varotsis C., Vamvouka M. (2002). J. Phys. Chem. B.

[cit15] Adamou A., Manos G., Messios N., Georgiou L., Xydas C., Varotsis C. (2016). Bioresour. Technol..

[cit16] Adamou A., Nicolaides A., Varotsis C. (2019). Miner. Eng..

[cit17] Jehlička J., Edwards H. G. M., Osterrothová K., Novotná J., Nedbalová L., Kopecký J., Němec I., Oren A. (2014). Philos. Trans. R. Soc., A.

[cit18] Llansola-Portole M. J., Pascal A. A., Robert B. (2017). J. R. Soc., Interface.

[cit19] Stavrakis S., Pinakoulaki E., Urbani A., Varotsis C. (2002). J. Phys. Chem. B.

[cit20] Romera-Castillo C., Sarmento H., Alvarez-Salgado X. A., Gasol J. M., Marrase C. (2010). Limnol. Oceanogr..

[cit21] Shimotori K., Watanabe K., Hama T. (2012). Aquat. Microb. Ecol..

[cit22] Blatt A., Lohr M. (2017). Bio-Protoc..

[cit23] Blatt A., Bauch M. E., Pörschke Y., Lohr M. (2015). Plant J..

[cit24] Giotta L., Mastrogiacomo D., Italiano F., Milano F., Agostiano A., Nagy K., Valli L., Trotta M. (2011). Langmuir.

[cit25] Ha J., Gélabert A., Spormann A. M., Brown G. E. (2010). Geochim. Cosmochim. Acta.

[cit26] Mikutta R., Baumgärtner A., Schippers A., Haumaier L., Guggenberger G. (2012). Environ. Sci. Technol..

[cit27] Kang S., Bremer P. J., Kim K., Mcquillan A. J. (2006). Langmuir.

[cit28] Tschirner N., Schenderlein M., Brose K., Schlodder E., Mroginski M. A., Thomsen C., Hildebrandt P. (2009). Phys. Chem. Chem. Phys..

[cit29] Alwis D. D. D. H., Chandrika U. G., Jayaweera P. M. (2015). J. Lumin..

[cit30] Kouki M., Hashimoto H., Kouama Y. (1990). Chem. Phys. Lett..

[cit31] Nelson N. B., Siegel D. A. (2013). Annu. Rev. Mar. Sci..

[cit32] Tourney J., Ngwenya B. T. (2014). Chem. Geol..

[cit33] Xiao N., Jiao N. (2011). Appl. Environ. Microbiol..

[cit34] Sun L., Xu C., Zhang S., Lin P., Schwehr K. A., Quigg A., Chiu M., Chin W., Santschi P. H. (2017). Chemosphere.

[cit35] Zhang Z., Chen Y., Wang R., Cai R., Fu Y., Jiao N. (2015). PLoS One.

